# Angiolipoma of the Lower Lip: A Rare Case Report

**DOI:** 10.7759/cureus.36875

**Published:** 2023-03-29

**Authors:** Ahmed A Dilli, Alaa A Abujabel, Ahlam Alharbi

**Affiliations:** 1 General Practice, Jouf University, Al-Jouf, SAU; 2 General Practice, Umm Al-Qura University, Makkah, SAU; 3 Family Medicine, Primary Health Care Center, Riyadh, SAU

**Keywords:** case report, oral cavity, lips, soft tissue tumours, angiolipoma

## Abstract

Angiolipoma is a benign soft tissue tumor composed of mature adipocytes and small capillaries. Although it can occur in any part of the body, it is rare in the oral cavity, especially in the lips. We report the case of a 47-year-old male who presented with a painless, slow-growing mass on the right lower lip that had gradually increased in size over several months. The initial diagnosis included lipoma, mucocele, and hemangioma. The mass was surgically excised, and the histological examination confirmed the diagnosis of angiolipoma. Angiolipomas involving the lips are rare and can be easily misdiagnosed. Therefore, clinical suspicion, along with appropriate imaging and histological examination, is essential for an accurate diagnosis and appropriate management. The patient had a smooth recovery after surgery and showed no recurrence of the condition during the one-year follow-up examination.

## Introduction

Angiolipoma is a rare, benign soft tissue tumor composed of mature adipocytes and small capillaries [1]. Although it can develop in any part of the body, its occurrence in the oral cavity, especially in the lips, is exceptionally uncommon, with only a few cases reported in the literature [2]. These lesions are typically small, painless, and slow-growing, ranging in size from a few millimeters to a few centimeters [3]. Nonetheless, their location in a highly visible area can cause concern for both patients and healthcare providers. Despite their benign nature, surgical excision is often the preferred treatment option [4]. In this case report, we present a case of angiolipoma involving the lower lip that was effectively treated with surgical excision.

## Case presentation

A 47-year-old male presented to the oral and maxillofacial surgery clinic with a painless, slow-growing mass on the right lower lip that had been present for several months. The patient reported that the lesion had gradually increased in size and was causing some discomfort with talking and eating. There was no history of trauma, bleeding, or infection. The patient had no significant medical history and was not taking any medications.

**Figure 1:**
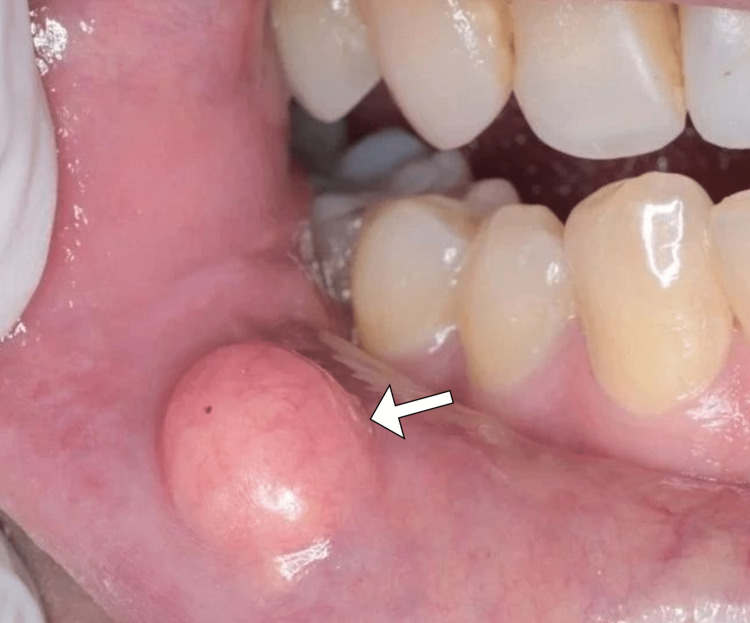
Clinical photograph showing a lower lip lesion with intact mucosa.

On clinical examination, a soft, compressible, non-tender submucosal mass was palpated on the right lower lip, measuring approximately 1.5 cm in diameter. The overlying mucosa was normal in color and texture, and there were no signs of inflammation or ulceration. The lesion was mobile and not attached to the underlying bone or surrounding soft tissues (Figure 1). The patient had no palpable cervical lymphadenopathy.

**Figure 2:**
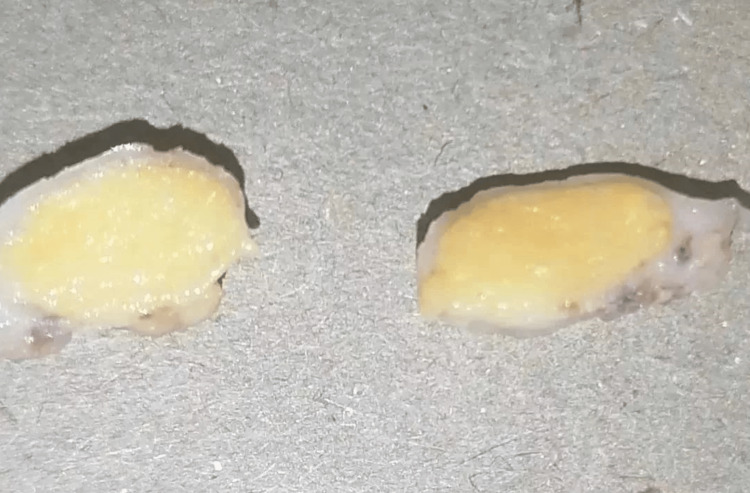
Gross photograph of the excised lesion, revealing a yellowish mass and supporting the diagnosis of a lipomatous lesion.

Based on the clinical presentation, the initial differential diagnosis included a lipoma, mucocele, and hemangioma. The patient underwent surgical excision of the lesion under local anesthesia. A transverse incision was made through the mucosa overlying the lesion, and the mass was carefully dissected from the surrounding tissues. The lesion was found to be a soft, yellowish, lobulated mass, measuring approximately 1.5 cm in diameter, with a well-defined capsule (Figure 2). The lesion was easily removed with the preservation of the surrounding tissues. The wound was closed with 5-0 Vicryl sutures.

**Figure 3:**
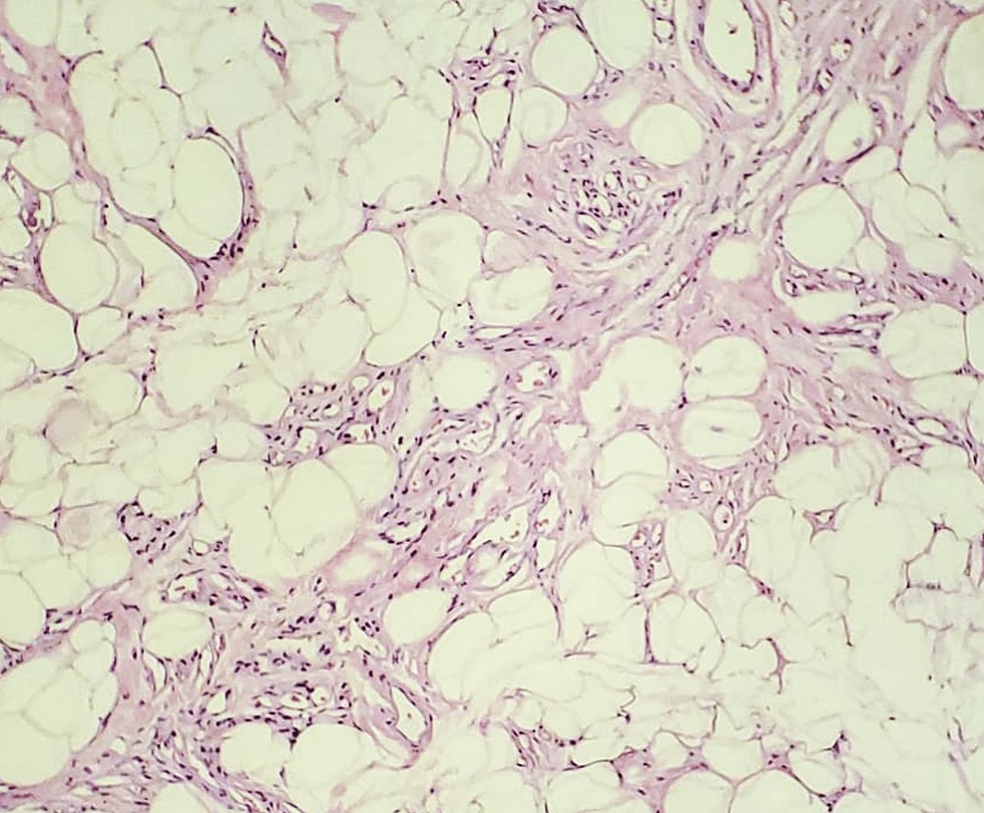
Histopathological image demonstrating adipose cells and interspersed capillaries, which confirms the diagnosis of angiolipoma.

The excised specimen was sent for histopathological examination, which revealed a well-circumscribed lipoma with no evidence of malignancy (Figure 3). The patient had an uneventful recovery, and the postoperative course was marked by mild pain and swelling, which resolved within a week. The patient was advised to avoid smoking and alcohol consumption and to maintain good oral hygiene. A follow-up visit was scheduled for two weeks post-surgery to monitor the healing process. At a one-year follow-up, there was no evidence of recurrence, and the cosmetic outcome was excellent.

## Discussion

Angiolipomas are rare tumors that are composed of mature adipose tissue and small capillaries. These tumors are generally slow-growing, painless, and small in size, ranging from a few millimeters to a few centimeters [1]. While angiolipomas can occur in any part of the body, they are exceptionally uncommon in the oral cavity [2]. Moreover, their occurrence on the lips is even rarer, and only a few cases have been reported in the literature [3,4].

In the present case, the diagnosis of angiolipoma was confirmed by examining the excised tissue histologically [1]. The histological features of angiolipoma typically include mature adipocytes interspersed with small capillaries [1]. This is in contrast to lipomas, which are composed solely of mature adipose tissue, and hemangiomas, which are composed of blood vessels.

The differential diagnosis for soft tissue masses in the oral cavity includes a wide range of benign and malignant conditions [2]. Among them, lipomas are the most common soft tissue tumors in the oral cavity, followed by fibromas, mucoceles, and hemangiomas [2]. The clinical presentation and radiographic findings, when available, can provide clues to the underlying pathology. However, a definitive diagnosis requires a histological examination of the excised tissue.

Surgical excision is the preferred treatment option for angiolipomas [3]. These tumors are typically well-defined, encapsulated masses that can be easily removed with the preservation of the surrounding tissues [3]. The surgical approach depends on the size and location of the lesion, as well as its adherence to the underlying bone or soft tissues [4]. In some cases, a more extensive surgical approach may be necessary to ensure the complete removal of the lesion [2,3].

Although angiolipomas are benign tumors, recurrence can occur after incomplete excision [4]. Long-term follow-up is therefore recommended to monitor for any signs of recurrence or malignant transformation [3]. In the present case, the patient had an uneventful postoperative recovery, and at a one-year follow-up, there was no evidence of recurrence. The cosmetic outcome was also excellent, which is particularly important for lesions that occur in highly visible areas such as the lips.

## Conclusions

Angiolipomas involving the lips are rare, benign soft tissue tumors that can be easily misdiagnosed. Clinical suspicion, along with appropriate imaging and histological examination, is essential for an accurate diagnosis and appropriate management. Surgical excision is the most common treatment, and complete excision with care to preserve surrounding tissues can result in excellent cosmetic outcomes.
